# Benchmark study for evaluating the quality of reference genomes and gene annotations in 114 species

**DOI:** 10.3389/fvets.2023.1128570

**Published:** 2023-02-21

**Authors:** Sinwoo Park, Jinbaek Lee, Jaeryeong Kim, Dohyeon Kim, Jin Hyup Lee, Seung Pil Pack, Minseok Seo

**Affiliations:** ^1^Department of Computer and Information Science, Korea University, Sejong City, Republic of Korea; ^2^Department of Computer Convergence Software, Korea University, Sejong City, Republic of Korea; ^3^Department of Food and Biotechnology, Korea University, Sejong City, Republic of Korea; ^4^Department of Biotechnology and Bioinformatics, Korea University, Sejong City, Republic of Korea

**Keywords:** reference genome, gene annotation, quality assessment, transcript diversity, next-generation sequencing (NGS), RNA-sequencing (RNA-seq), livestock animals, model organisms

## Abstract

**Introduction:**

For reference genomes and gene annotations are key materials that can determine the limits of the molecular biology research of a species; however, systematic research on their quality assessment remains insufficient.

**Methods:**

We collected reference assemblies, gene annotations, and 3,420 RNA-sequencing (RNA-seq) data from 114 species and selected effective indicators to simultaneously evaluate the reference genome quality of various species, including statistics that can be obtained empirically during the mapping process of short reads. Furthermore, we newly presented and applied transcript diversity and quantification success rates that can relatively evaluate the quality of gene annotations of various species. Finally, we proposed a next-generation sequencing (NGS) applicability index by integrating a total of 10 effective indicators that can evaluate the genome and gene annotation of a specific species.

**Results and discussion:**

Based on these effective evaluation indicators, we successfully evaluated and demonstrated the relative accessibility of NGS applications in all species, which will directly contribute to determining the technological boundaries in each species. Simultaneously, we expect that it will be a key indicator to examine the direction of future development through relative quality evaluation of genomes and gene annotations in each species, including countless organisms whose genomes and gene annotations will be constructed in the future.

## Introduction

Next-generation sequencing (NGS) technology is applied in many ways to identify the biological characteristics of various organisms, including livestock, at the molecular level (1, 2). This technology is used in virtually all biomedical fields, such as research to find genetic variants based on DNA sequencing (3, 4) and research to discover transcripts related to life phenomena based on RNA-sequencing (RNA-seq) (5, 6). Recently, NGS technology has been developed for data acquisition of molecular characteristics at the level of single cells (7) or single nuclei (8), concurrently, long-read-based technologies are continuously being developed to improve sequencing quality (9). Various technologies are continuously being developed to measure various levels of molecular markers more accurately; however, all of them are strongly dependent on the reference genome and gene annotation corresponding to the biological species of the targeted subject in certain studies (10). As of 2023, the fundamental and essential data of the NGS technique, reference genomes and gene annotations, have been established in the Ensembl database for 314 species (11). Moreover, it is highly likely that the number of completed reference genomes and gene annotations for more species will increase exponentially in the near future through the vertebrate genome project (VGP) (12). Thus, a relative comparison of relevant essential data is necessary to increase the reliability of various applied studies in more diverse species. Although the accuracy of the results of each study utilizing NGS highly depends on the completeness of the two key underlying data, there has been no systematic evaluation of reference genomes and gene annotations among diverse species simultaneously. Although, species have a common genetic background, to some extent, the genome structure, number, and type of transcripts differ considerably between organisms, which makes comparisons across species quite challenging (13, 14).

To date, various attempts have been made to identify the whole-genome sequence in a particular species by selecting the optimal assembly from a number of draft assemblies. Various methodologies such as, KAT (15), Merqury (16), and Inspector (17), have been developed to compare the quality of different versions of draft assemblies for a specific target species to determine a representative genome. However, these methodologies require whole genome sequencing (WGS) reads and/or a reference genome of the target species, therefore, they cannot be directly applied for the purpose of evaluating the quality of reference genomes for multiple species. Among these tools, BUSCO (18, 19) can be used to compare the quality of reference genomes for multiple species based on the orthologous genes. However, since the optimal assembly was already determined in the direction of optimizing the BUSCO completeness in the process of completing the reference assembly of each species, the difference in BUSCO completeness of the published reference genome is very small among species. Although we currently lack systematic methodologies that can be used to directly and simultaneously compare the quality of reference assemblies of various species, some indicators can be used to compare species. First, the quality of the reference genome was compared using a contiguity index, such as the N50 value obtained based on the relative length of contigs or scaffolds generated during the *de novo* assembly process (20–22). Another quality evaluation index for the completed genome is the number and frequency of gaps in the genome, and various attempts have been made to reduce them (23–25). However, gene annotation quality assessment methods remain poorly understood, owing to their transcriptome diversity. Recently, software has been developed that can estimate the annotation similarity of evolutionarily adjacent species based on the gene annotations of species known to be nearly complete, allowing a relative comparison of the gene annotations of the two species (26). However, there is, to date, no known systematic approach to compare gene annotations of multiple species.

Although long-read sequencing technology is continuously being refined, NGS application research is still mainly based on short-read sequencing technology. RNA-seq, a representative application of NGS based on short reads, generally involves a two-step analysis. The first step is an alignment process to determine where the short-read fragmented sequences originate from the genome, for which the quality of the reference genome is important (27, 28). If the accuracy of the sequence of the reference genome is low, the mapping rate is directly affected. If the frequency of repeat sequences is high, the number of multiple mapping reads increases, adversely affecting the entire process. The second major step for processing RNA-seq data is to quantify the mapped reads in the genome (29). At this time, performance greatly depends on the quality of the gene annotation, which defines the location of the transcripts in the genome (30, 31). If all transcripts that can occur in a specific organism are included in gene annotation, the quantification rate will increase; however, the probability of overlapping other transcripts at a specific genome location will correspondingly increase, resulting in quantification failure due to ambiguity. Concurrently, inclusion of transcripts that are too conservative in gene annotations to address this ambiguity exacerbates quantification failures caused by the absence of annotations. These issues are commonly considered when developing reference genomes and gene annotations for various species, thus the quality of the two fundamental types of data can be measured indirectly through the corresponding indicators at the alignment and quantification steps.

Based on these rationales, in this study, we attempted to evaluate the quality of reference genomes and gene annotations of all species as much as possible, which has not yet been performed because of technical issues. We attempted to measure the quality of two key data essential in NGS from various angles by assessing the effectiveness of new potential indicators along with the indicators that have been used so far for quality evaluation. In addition, we aimed to demonstrate a new integrated index for the simultaneous quality evaluation of genome and gene annotation, by applying selected quality effective indicators to RNA-seq data derived from various species.

## Materials and methods

### Reference genome and gene annotation collection

As of November 2022, the latest genome assembly (.fasta) of each species and the corresponding gene annotation (.gtf) were collected from the Ensembl database (Supplementary Table 1) using Rcurl v1.98.1. Among all species, human, mouse, and zebrafish species that had access to the primary assembly version were used, and the toplevel version of the genome was used for the rest of the species.

### Collection of basic statistic on genome assembly and gene annotation

Basic assembly information for all species was collected in xml format through the API of ENA (European Nucleotide Archive) (https://www.ebi.ac.uk/ena/browser/api/xml/Assembly accession). The collected assembly basic statistics were tabulated using xml2 (v1.3.3) and tidyverse (v1.3.2) R packages. We also collected detailed information on gene annotation from Ensembl biomart (32) using the biomaRt (v2.50.3) R package. Using the getBM function, various information including ensemble gene id and gene type were collected and tabulated from the gene annotation of each species. The transcript types in gene annotation were classified into 30 types according to the classification criteria of Ensembl gene biotype (https://asia.ensembl.org/info/genome/genebuild/biotypes.html) (Supplementary Table 2).

### Estimation of repeat elements from reference genomes

The Repeat Masker (v4.1.4) (33) with -pa 16 -qq options was used to quantify repeat elements from reference genomes of various species. RMBlast (v2.11.0) was used as the repetitive sequence search algorithm, and the search was based on the Dfam (v3.6) database (34). In addition, TRF (v4.09) (35) was used to find tandem repeat sequences.

### RNA-seq raw data collection

As of November 2022, among the species whose reference genome and gene annotation are listed in the Ensembl database, we searched for species that could secure RNA-seq data of more than 30 samples. Using R (v4.1.2) language-based packages XML (v3.99.0.12) and xml2 (v1.3.3), data corresponding to the following conditions was retrieved from NCBI Esearch (https://eutils.ncbi.nlm.nih.gov/entrez/eutils/esearch.fcgi) and 30 SRA IDs of each species were randomly selected. In XML parsing with the GET method, we consider the following four conditions: “biomol rna”, “library layout paired”, “platform illumina”, and “Bulk”. After that, we used the prefetch (v2.11.2) included in the SRAtoolkit (v2.11.3) to import randomly selected sra files from the SRA database (36). To convert the collected sra files into paired-end fastq format files, parallel-fastq-dump was employed. The FastQC v.0.11.9 (37) was used to check the quality of the collected raw sequencing data.

### Preprocessing of RNA-seq data

All collected genomes were indexed using the full Hisat2-build (v.2.2.1) (38). Paired-end RNA-seq files whose quality was checked through FastQC (v.0.11.9) were mapped to each corresponding genome. Alignment results were recorded in sorted bam format through samtools view (v1.14), and mapping-related statistics were collected through samtools stats. The mapped reads to each genome were quantified using featureCounts (v2.0.1) (39) with the corresponding gene annotation.

### Quality evaluation indicators for reference genome in diverse species

A total of 10 indicators used in this study are summarized in Table 1. All indicators are scaled in the range of 0–1 for the convenience of interpretation. Also, the closer the value is to 1, showing the better the quality in all indicators.

**Table 1:** Selected 10 indicators for quality evaluation of reference genome and gene annotation in diverse species.

**No**.	**Abbreviation of indicators**	**Description**	**Category**	**Formula**	**Scaling method**	**Range of values**
1	AdjN50Contig	Percentile of adjusted N50 by genome size in contig	Assembly stat.	N50 value in contigs/genome size	Percentile	0–1
2	AdjN50Scaffold	Percentile of adjusted N50 by genome in scaffold	Assembly stat.	N50 value in scaffolds/genome size	Percentile	0–1
3	UngapRate	Scaled non-spanned gaps rate	Assembly stat.	1—[spanned gaps/max (spanned gaps)]	NA	0–1
4	UnimapRate	Uniquely mapped rate	Mapping stat.	Uniquely mapped reads/total # of reads	NA	0–1
5	MapRate	1—unmapped reads' rate	Mapping stat.	1—(unmapped reads/total # of reads)	NA	0–1
6	MultiMapRate	1—multiple mapped rate	Mapping stat.	1—(multiple mapped reads/total # of reads)	NA	0–1
7	Transcript diversity	Scaled transcript diversity calculated by PCA	Gene annotation	PC1 obtained from PCA analysis	{X—min (X)}/{max(X)—min (X)}	0–1
8	Quant.rate	Quantification success rate from the mapped reads on the genome	Quantification stat.	Quantification success reads/total # of mapped reads	NA	0–1
9	Quant.rate (Abs)	1—quantification failure rate due to absence of annotation	Quantification stat.	1—(unquantified mapped reads due to absence of annotation/total # of mapped reads)	NA	0–1
10	Quant.rate (Amb)	1—quantification failure rate due to ambiguity	Quantification stat.	1—(unquantified mapped reads due to ambiguity / total # of mapped reads)	NA	0–1

All indicators are scaled to have a value between 0 and 1 and the closer each index value is to 1 represents the better quality.

As indicators for simultaneous relative evaluation of the genomes of various species, three indicators were selected based on the statistics derived from the assembly process. Based on the N50 values of contig and scaffold, which are the continuity indices of assembly, it was corrected to consider the different genome size of various species. These corrected N50s were converted to have a range of 0 to 1 by their percentile. Through this, two indicators, *AdjN50Contig* and *AdjN50Scaffold*, were calculated respectively. Next, to get the *UngapRate*, it was subtracted from 1 to adjust the directionality after obtaining the ratio of spanned gaps in the genome of each species compared to the species with the largest spanned gaps among all species.

We selected three empirical indicators obtained through the process of mapping actual NGS data as another measure to evaluate the quality of the genome. First, *UnimapRate* is basically the most important indicator in the mapping step, and represents the ratio of reads uniquely mapped to a specific genomic region among all reads. In addition, we additionally considered the two typical causes of mapping failure: multi-region mapping and no corresponding region. To match the direction as a quality evaluation index, *MapRate* and *MultiMapRate* indexes were constructed by subtracting the two failure rates from 1, respectively. Based on these three empirical indicators, we construct a new mapping quality evaluation index (MQI) for species *i*:


(1) $$MQI_{i}=\;(UnimapRate{\;}_{i}+\;MapRate{\;}_{i}+\;MultiMapRate{\;}_{i})\;/\;3$$

The MQI_i_ is the arithmetic mean of the three different directional indices obtained empirically from the mapping step, and is a relatively comparable indices across different species. Additionally, the BUCSO completeness was calculated using BUSCO (v5.4.2) with–auto—lineage-euk–cpu 16 options (18).

### Quality evaluation indicators for gene annotation in diverse species

To qualitatively evaluate the quality of gene annotation, the proportion of each gene type was calculated based on the gene types collected from Ensembl biomart (32). Based on a matrix with a total of p gene type ratios for all species n, principal component analysis (PCA) was applied that can secure a linear combination of p gene type ratio random variables to convert to a nx1 vector for comparing all species n. After examining the degree of the variance explain based on the eigen values, the PC1 embedding values were extracted and used as *Transcript diversity*. Additionally, to further clarify the interpretation of PC1, another method of summarizing variability, Shannon's equability index (40, 41), was calculated and compared.

As another criterion for evaluating the quality of the gene model, we selected three empirical indicators obtained through the process of quantifying reads mapped to the genome based on actual NGS data. First, *Quant.rate*, which is the ratio of reads successfully quantified as gene counts among mapped reads derived from each sample, was selected with the highest priority. Simultaneously, the absence and ambiguity of annotation, which are two representative quantification failure rate factors that can be determined by the gene model, were additionally considered. To match the directionality, two indicators, *Quant.rate (Abs)* and *Quant.rate (Amb)*, were set by subtracting the two failure rates from 1. Based on the three empirical indices obtained during the quantification process, we constructed the comprehensive quantification quality evaluation index (QQI) for species *i*:


(2) $$QQI_{i}=\;(Quant.rate_{i}+\;Quant.rate{\left(Abs\right)}_{i}+\;Quant.rate{\left(Amb\right)}_{i})\;/\;3$$

The QQI_i_ is the average of the three indices obtained empirically in the quantification stage of NGS data and is an indicator that can simultaneously compare the general quality of gene models in multiple species.

### NGS applicability index

Based on a total of 10 effective indicators that can evaluate the genome and gene model (Table 1), it was generalized as an index representing the technical boundary of NGS technology in a specific species. The formula consisting of the weighted arithmetic mean of the 10 indicators for each species *i* is:


(3) $$\begin{array}{l} NGS\;applicability\;index_{i}=\\ \frac{w_{1}AdjN50Contig_{i}+\;w_{2}AdjN50Scaffold_{i}+\ldots +\;w_{10}Quant.rate{\left(Amb\right)}_{i}}{\sum_{i=1}^{10}w_{i}} \end{array}$$

In this study, all 10 weights w1, w2, …, w10 were considered as 1, which means that all indicators are considered equally.

## Results

### Large-scale NGS data collection for quality evaluation of reference genomes and gene annotations of 114 species

**Figure 1:**
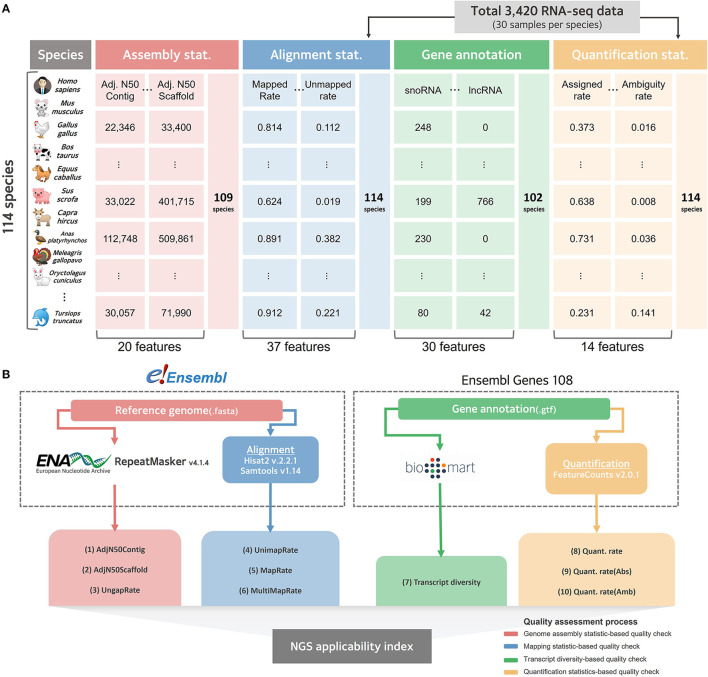
Collected data structure and schematic diagram for benchmarking comparison. **(A)** Overall structure of data collected for quality evaluation of reference genome and gene annotation for 114 species. **(B)** A systematic workflow to select effective indexes for relative quality assessment from collected data. The red line represents a pipeline that selects effective indicators from assembly statistics for relative evaluation of the reference genome. The blue line represents the process of empirically evaluating the quality of the genome by mapping the actual NGS data in the alignment step. The green line represents the process of calculating the transcript diversity index, and the yellow line represents the pipeline that empirically finds effective indicators for quality evaluation of gene annotations in the quantification process.

We systematically collected data to evaluate the current level of reference genomes and gene annotations for as many species as possible, for which RNA-seq, among various NGS technologies, could be directly applied (Figure 1A). There were more than 30 publicly available RNA-seq datasets for 114 of the 314 species (Supplementary Table 1), whose reference genomes and gene annotations are listed in the Ensemble database (11). As a result of organizing the taxonomic categories for these 114 species compared in this study, it was confirmed that 1 fungus, 112 Metazoa, and 1 Viridiplantae were included at the kingdom level (Supplementary Table 3). At the taxonomic level, they were classified into 11 types, of which 47 Actinopteri, 43 Mammalia, and 12 Aves were the majority. In addition to collecting the latest version of the reference genome and gene annotation for these 114 species, 30 RNA-seq datasets per species were randomly collected, resulting in a total of 3,420 RNA-seq datasets (Supplementary Table 4). After the quality check, an average of 34 million reads and an average Phred score of 36.237 were observed, showing no technical issues in the collected RNA-seq data (Supplementary Table 5). When the collected RNA-seq data were mapped based on the reference genome representing each species, an average overall alignment rate of 84.768% was obtained (Supplementary Table 6). In quantification step, 55.807% of mapped reads were successfully quantified to genes in average (Supplementary Table 7).

To independently compare the quality of all 114 collected reference genomes, genome assembly statistics were compiled from the European Nucleotide Archive (42) and the corresponding information was missing for five species. The remaining 109 available species were systematically collected, and assembly related statistics were obtained from the collected data, resulting in an average length of 1,689,594,967 bp and a contig average N50 of 7,154,707 bp (Supplementary Table 8). We also collected data from Ensembl Biomart (32) to evaluate the quality of gene annotations that indicated the location of genic regions in the reference genome of each species; however, the information could not be collected for 12 out of 114. For the remaining 102 species, gene annotation was collected and classified as a total of 30 types of RNAs, including long non-coding RNA (lncRNAs) and microRNAs (miRNAs) (Supplementary Table 9). We found that an average of 22,915 protein-coding genes were annotated across all 102 species, while a significantly small number of average 2,340 lncRNAs were not annotated in 37 species.

Based on the collected data at various levels, an experimental design was established that measures the quality of the genomes and gene models in various species (Figure 1B). In this current study, we focused on quality measures for eight species of livestock designated according to the Food and Agriculture Organization of the United Nations (FAO).

### Comparison of assembly statistics and frequency of repeat elements for reference genome quality evaluation in 109 species

**Figure 2:**
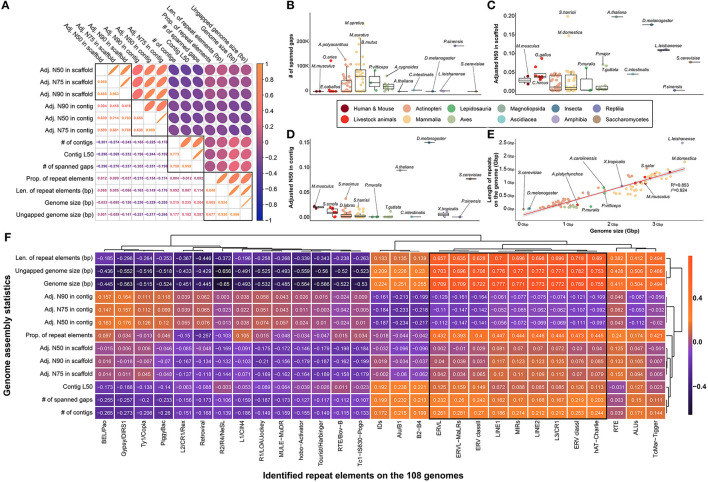
Comparison of assembly statistics for selection of effective indicators for genome quality evaluation of various species relatively. **(A)** Investigation of correlation between various assembly statistics and repeat elements that are presumed to be related to genome quality evaluation. Pearson's correlation coefficients were used to pairwise investigation. Four major types of indicators showed strong correlations. **(B)** The number of spanned gaps remaining in the genomes of 109 species. **(C)** Comparison of adjusted N50 in scaffold level by genome size. **(D)** Comparison of adjusted N50 in contig level by genome size. **(E)** Strong correlation between genome size and total length of repeat elements in each species. R^2^ and r^2^ represents coefficient of determination and correlation coefficient, respectively. **(F)** Correlation between the assembly statistics and the amount of various types of repeat elements found in the genome of 108 species. **(B–E)** The colors in the figure share group information separated by class taxonomic level, except for human-mouse and eight livestock animals.

Officially published reference genomes of various species are generally expected to show minimal difference in quality owing to the robustness of DNA; however, limitations exist due to the frequency of repetitive sequences in the genome and/or sequencing technology based on short reads. To investigate this, we collected and compared representative quality statistics of 109 genome assemblies, which were largely clustered into four characteristics (Figure 2A). While an average of 37,580.454 spanned gaps were found in all species, only 204 and 661 spanned gaps were found in the human and mouse genomes, respectively, which are known to be of high quality (Figure 2B). In addition, a significantly lower number of spanned gaps was observed in representative model organisms such as *Saccharomyces cerevisiae* (*S.cerevisiae*), *Arabidopsis thaliana* (*A.thaliana*), and *Drosophila melanogaster* (*D.melanogaster*) (Figure 2B, Supplementary Figure 1). While a low number of spanned gaps was found in most of the eight livestock animals, it was confirmed that a relatively large number of spanned gaps were present in the genomes of *Ovis aries* (125,067 gaps) and *Equus caballus* (6,286 gaps).

Furthermore, we found that the number of spanned gaps was strongly correlated with the number of contigs generated during the *de novo* assembly process, which revealed that in the case of species with many spanned gaps, relatively short contigs occurred during the assembly process (Figure 2A, Supplementary Figure 2). In other words, various technical issues derived from short sequence read assembly intensify depending on the number of spanned gaps ultimately affecting the quality of the completed genome assembly, which suggests that the genome quality of various species can be evaluated based on these statistics. Further evidence for this claim can be found in the negative correlations between the number of spanned gaps and adjusted N50, N75, and N90 values by genome size in both contigs and scaffolds (Figure 2A). These values are representative indicators used when evaluating the quality of the genome completed through *de novo* assembly, and significantly higher values were observed in representative model organisms at both the scaffold and contig levels (Figures 2C, D). It was confirmed that at least one model animal in representative species at each class taxonomic level, such as yeast, Drosophila, chicken, and frog, has an extremely high complete genome.

Since various types of repeat elements widely spread across the genome are a representative cause of difficulty in the genome assembly process, we further investigated the frequency of repeat sequences in the genome of each species to evaluate the quality of each reference genome. We hypothesized that genome repeat frequencies in each species could help assess the quality of the reference genomes; however, there was no association with various genome quality indicators (Figure 2A). We found that one of the primary reasons for this observation is that the genome size varies across species, depending on the class taxonomic level, and that genome size determines the types of repeat elements that can be found (Figure 2E). A correlation of 0.924 was observed between the length occupied by all repeat elements in the genome and the length of the genome, supporting this claim. In addition, it is further evidence that the length of the region occupied by the repeat sequence in the entire genome is mostly dependent on long repeat sequences such as LINE1 and LINE2 (Figure 2F). Although all species had a consistent linear pattern in their genome size and ratio of repeat elements, we found that species such as *Leptobrachium leishanense* had a high ratio of repeat elements to genome size (Figure 2E). However, since we cannot be sure whether these results are due to the characteristics of the genome of the species, we ultimately concluded that it is difficult to use the ratio of repeat elements as an effective measure to evaluate the quality of the genome. Additionally, we used BUSCO to compare the quality of reference assemblies of multiple species based on the orthologous genes. In result, all BUSCO completeness in 109 species had high values (97.255 in average) with no significant differences, which means that there is no value as an effective indicator for comparing multiple species with reference genomes (Supplementary Figure 3).

### Demonstration of change in the mapping quality of RNA-seq data according to the completeness of the reference genome

**Figure 3:**
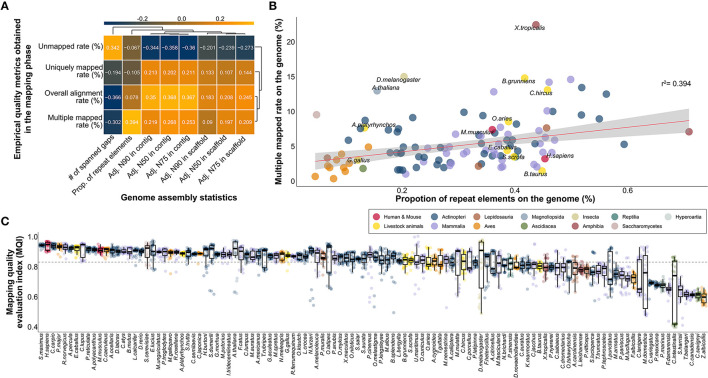
Investigation of association relationship between assembly statistics and empirical effective indicators obtained in the mapping step for genome quality evaluation. **(A)** Pairwise correlation between selected assembly statistics and empirical effective indicators obtained in the mapping step for genome quality assessment. **(B)** Linear relationship between the ratio of multiple mapped reads and proportion of repeat elements on the genome in 108 species. r^2^ represents correlation coefficient, respectively. **(C)** Differences in newly proposed MQI values in 108 species. To evaluate the relative quality of the genome, valid empirical indicators were integrated and configured in the mapping stage. The horizontal line represents the average MQI value across all 108 species.

We demonstrated whether the representative indicators used to evaluate the quality of the reference genome affect the mapping step of RNA-seq data processing. For the remaining 108 species, excluding *Salmo trutta*, for which repeat elements were not identified among 109 species, 3,240 RNA-seq data were mapped to their corresponding reference genome in a non-repeat masked version. Although no clear linear relationship was observed when the characteristics of different species were considered simultaneously, we found that the mapping failure rate increased, and the unique mapping and total alignment rates decreased as the number of spanned gaps increased (Figure 3A). Similarly, in another assembly contiguity index, with N50, N75, and N90 adjusted by genome size, it was demonstrated that the mapping failure rate decreased, and the mapping success rate increased when longer contig or scaffold values were observed. These results provide evidence that the quality of the mapping step is directly affected by the genome completeness.

We also demonstrated that the multiple mapping problem intensifies depending on the ratio of the repeat elements in the genome. It was demonstrated that the rate of multiple mapping reads increased (*r*^2^:0.394) in genomes with a high frequency of repetitive sequences across all species (Figures 3A, B). This is because the genome used in this experiment was an unmasked version of the repeat elements. If the genome utilized repetitive masked versions commonly used in RNA-seq, the multiple mapping rate would not increase, but the overall mapping rate would decrease. The average multi-mapping rate in all species was 5.68%, whereas a multi-mapping rate of 22.512% was observed in *Xenopus tropicalis*. High multi-mapping rates were also observed in model organisms such as *D. melanogaster* (15.068%) and *A. thaliana* (13.034%). These results demonstrate that multi-mapping of reads intensifies according to the ratio of repeat sequences in the genome, however this could be because of the characteristics of the species, not the quality of the genome (Figure 3B).

Finally, we compared all species with MQI based on valid indicators generated in the mapping step. An average MQI of 0.829 was observed across all species, indicating that there are very few species with genomes that perform poorly enough to affect mapping in most publicly available reference genomes (Figure 3C).

### Qualitative evaluation of gene annotations from 102 species through comparison of transcript diversity

**Figure 4:**
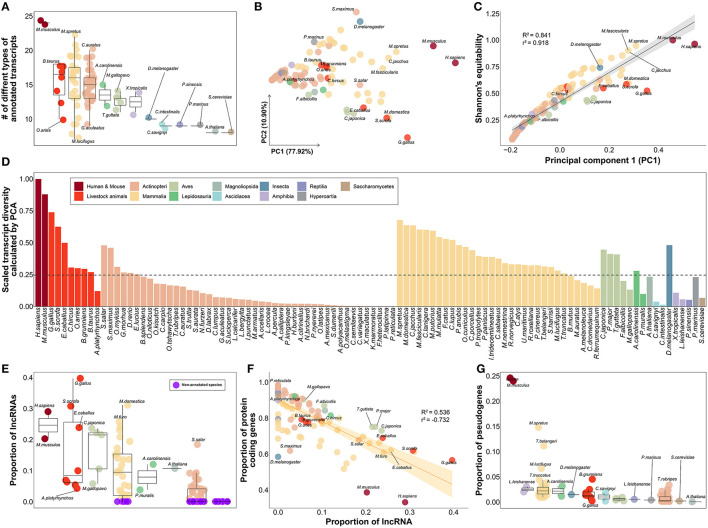
Qualitative evaluation of gene annotation based on transcript diversity. **(A)** Comparison of the number of types found in each species out of a total of 30 transcript types annotated in gene annotations of 102 species. **(B)** Dimensional reduction results for the ratio of 30 transcript types in gene annotation of each species through principal component analysis. About 77.92% of the total variance in the original data was explained by the first principal component. **(C)** Results of correlation investigation between two methods of estimating transcript diversity: Shannon's equitability and PC1 obtained through PCA. R^2^ and r^2^ represents coefficient of determination and correlation coefficient, respectively. **(D)** Comparison of transcript diversity index for all 102 species. **(E)** Comparison of the proportion of annotated lncRNAs in the gene annotations of each species. Purple indicates species with no lncRNA annotated at all. **(F)** Correlation between the proportion of lncRNAs and the proportion of protein-coding genes. R^2^ and r^2^ represents coefficient of determination and correlation coefficient, respectively. **(G)** Comparison of the proportion of annotated pseudogenes in the gene annotations of each species. **(A–E)** The colors in the figure share group information separated by class taxonomic level, except for human-mouse and eight livestock animals.

Based on 30 different types of genes included in the gene annotation collected from a total of 102 species (Supplementary Table 9), we evaluated the relative level of gene annotation in various species, including livestock. We hypothesized that the gene annotations for humans and mice, which have been frequently and continuously revised through the efforts of many researchers, would be at the highest level. The fact that 24 of the 30 classification criteria of the transcript types in gene annotation were observed in human and mouse species demonstrates that this is the most subdivided gene annotation when compared to other species, as we hypothesized (Figure 4A). Therefore, it was further hypothesized that by measuring the transcript diversity of gene annotation within a specific species, it would be possible to measure the relative level of gene annotation of that species compared to humans or mice, which have relatively well-organized gene annotations.

As a result of investigating gene diversity in annotations using a dimensionality reduction algorithm based on the ratio of 30 different types of genes derived from 102 gene annotations, no species has yet reached the level of human or mouse gene annotation (Figure 4B). The PC1 values obtained from dimensionality reduction analysis explained 77.92% of the total transcript diversity in gene annotations, and the strong correlation with Shannon's equitability calculated based on mouse species supports our claim (Figure 4C). We evaluated the diversity of transcripts in each of the 102 gene annotations and found the highest diversity in human (Figure 4D). Based on human's transcriptome diversity, mouse gene annotation followed with 87.996%. In the case of mammals, the average diversity of gene annotations was generally higher than that of other classes. Livestock were confirmed to have approximately 39.463% diversity compared to that of the human gene annotation. Of the eight livestock species highlighted in this study, only 12.099% of the human gene annotation complexity was annotated in the mallard duck (*Anas platyrhynchos; A. platyrhynchos*). While gene annotations with more than 50% transcript diversity were rare in other classes, relatively high gene annotation diversity levels of 48.095% and 47.999% were found in *D. melanogaster* and *Salmo salar*, respectively.

We further investigated whether the transcript diversity index was significantly affected by which of the 30 transcript types (Supplementary Table 10). It was found that lncRNA had a correlation of 0.841 with the transcript diversity obtained from the dimensionality reduction analysis. We found that lncRNAs in 37 species, including *D. melanogaster*, were not classified in the annotation (Figure 4E, Supplementary Table 9). Among the 8 livestock animals, 11,944 and 10,965 lncRNAs were annotated in *Gallus gallus* and *Sus scrofa*, respectively. In contrast, relatively low numbers of 1,480 and 786 lncRNAs were annotated in *Bos taurus* and *A. platyrhynchos*. We presumed that protein-coding genes would contribute considerably to the diversity of gene annotation, but correlation of −0.126 with transcript diversity was found (Supplementary Table 10). In addition, the average proportion of protein-coding genes was 81.69% in all 102 species (Figure 4F). These results demonstrated that when constructing gene annotations across all species, protein-coding genes are usually annotated as primary targets; thus, they did not significantly contribute to the classification of the 102 species based on the diversity of transcripts within the annotations. However, we identified relatively low proportions of protein-coding genes in model organisms such as humans (33.041%), mice (38.538%), chickens (56.487%), and *D. melanogaster* (58.365%). Concurrently, we found that various small RNAs, such as small nuclear RNA (snRNA), small nucleolar RNA (snoRNA), small Cajal body-specific RNA (scaRNA), and miRNA, also play an important role in determining the level of transcript diversity for gene annotations in 102 species (Supplementary Figure 4). This implies that as non-coding genes other than protein-coding genes are included in the gene annotation, the proportion of protein-coding genes decrease, suggesting that this can be another indicator of the degree of development of gene annotation. Finally, we observed a correlation of 0.545 between transcript diversity and the ratio of pseudogenes (Supplementary Table 10). Excluding human and mouse gene annotations, the average proportion of genes classified as pseudogenes in the gene annotations of the remaining 100 species was only 1.523% (Figure 4G). In contrast, in humans and mice, a significant number of annotated genes were classified as pseudogenes, at 24.571 and 23.961%, respectively. This result indicates that the level of gene annotation is generally higher, as pseudogenes are additionally considered in gene annotation beyond the level of simple classification of protein-coding genes, lncRNAs, and some small RNAs whose functions are known or are of common interest to scientists.

### Demonstration of change in mapped reads quantification performance according to the quality of gene annotation in 102 species

**Figure 5:**
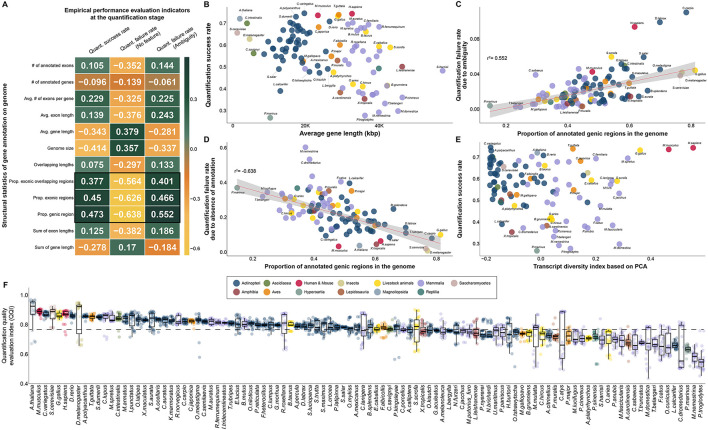
Selection of empirical effective indicators in the quantification process and investigation of correlation with complexity of transcripts in annotation for quality evaluation of gene annotation in diverse species. **(A)** Correlation between 12 characteristics of gene annotation and 3 quality evaluation indicators obtained empirically in the quantification process. **(B)** Scatter plot between average gene length and quantification success rate. **(C)** Association between the proportion of annotated genic regions in the genome and the rate of quantification failure due to ambiguity. **(D)** Correlation between the percentage of annotated genic regions in the genome and the rate of quantification failure due to the absence of annotation. **(E)** Independence between transcript diversity index, a proposed qualitative quality evaluation metric, and quantification rate, a quantitative quality evaluation index. **(F)** Differences in QQI, an empirical quality index obtained at the quantification stage, in all species. **(B–F)** The colors in the figure share group information separated by class taxonomic level, except for human-mouse and eight livestock animals.

Quantification of reads generated from RNA-seq data is a crucial process for measuring gene expression levels and is most frequently applied to various biomedical fields. In the process of quantifying the reads mapped to the genome, we speculated that the quantification success rate would be affected by the structure and completeness of the gene annotation of various species. Based on RNA-seq data from all 102 species, we found that the proportion of annotated exon (*r*^2^ = 0.45) or gene (*r*^2^ = 0.473) in the genome correlated most with the proportion successfully assigned to a specific gene during the quantification process (Figure 5A). We found patterns clearly differentiated by average gene length in 102 species at the class taxonomic level and identified the quality of gene annotation within each class in terms of the quantification rate for mapped reads on the genome (Figure 5B). For example, human (0.745) and mouse (0.738) gene models are of outstanding quality in mammals; however, the quantification rates were significantly low in *Macaca nemestrina* (0.293) and *Pan troglodytes* (0.279). High quantitative success rates were observed in *G. gallus* (0.734) and *A. thaliana* (0.722), which are representative model bird and plant species, respectively. However, in *Petromyzon marinus* (0.268), which represents the Hyperoartia class, it was confirmed that RNA-seq application research is not yet possible in terms of the quantification rates of mapped reads.

While genomic features were distinct for each class taxonomic level, we found a common pattern across 102 species in two representative causes of mapped reads for which quantification failed (Figures 5C, D). The first representative cause of quantification failures caused by gene annotation was ambiguity due to redundant annotations at genomic locations (Figure 5C). We demonstrated that a higher percentage of genes annotated in the genome of a particular species, led to increased ambiguity (*r*^2^ = 0.552) in the quantification step (Figures 5A, C). Interestingly, it was also found that human and mouse gene annotations, which had a high quantification success rate, were not free from redundancy problems, suggesting that short-read-based NGS technology continue to have difficulties in accurate quantification. We further investigated the absence of gene annotation, which is another representative cause of quantification failure for mapped reads caused by gene annotation. As a result, we identified a common pattern in which higher frequency of genes annotated in the genome, led to the lower quantification failure rate (*r*^2^: −0.638) due to the absence of annotation (Figure 5D). We demonstrated that in most model organisms, including humans (0.065) and mice (0.035), the rate of quantification failure caused by the absence of gene annotation was relatively low compared to that in other species. We also demonstrated that these two representative quantification errors (Figures 5C, D), caused by the characteristics of gene annotation, were opposed to each other in 102 species through actual RNA-seq data. For example, human and mouse annotations include annotations for many genes compared to other species, reducing errors due to the absence of annotations; however, errors due to redundancy of annotations are relatively high. In this regard, we additionally investigated the association between the diversity of annotated transcript types and the success rate of quantification, but no association was observed (Figure 5E). This result demonstrated that the transcript diversity index does not affect the quantification success rate index, as it does not affect the exon or gene structure in gene annotation. In addition, the transcript diversity index has been demonstrated to be another independent index that can evaluate gene annotation qualitatively in a different direction than the quantification success rate index.

We finally compared a QQI for 102 species based on the quantification success rate and two quantification failure rates, which are determined by the quality of gene annotation (Figure 5F). As a result, it was found that the average QQI was high in the order of *A. thaliana* (0.89), mouse (0.887), *C.variegatus* (0.871), *S.cerevisiae* (0.866) and chicken (0.863). This result demonstrates that most model organisms whose gene annotations have been frequently updated are of markedly high quality compared to other species through the quantification process with real 3,060 RNA-seq data from 102 species. In contrast, this suggests that there are still practical problems with accurate quantification due to quality problems of gene annotation in species belonging to Mammalia, such as *Camelus dromedarius* (0.644), *Macaca nemestrina* (0.585) and *Pan troglodytes* (0.557).

### Application and validation of NGS applicability index

**Figure 6:**
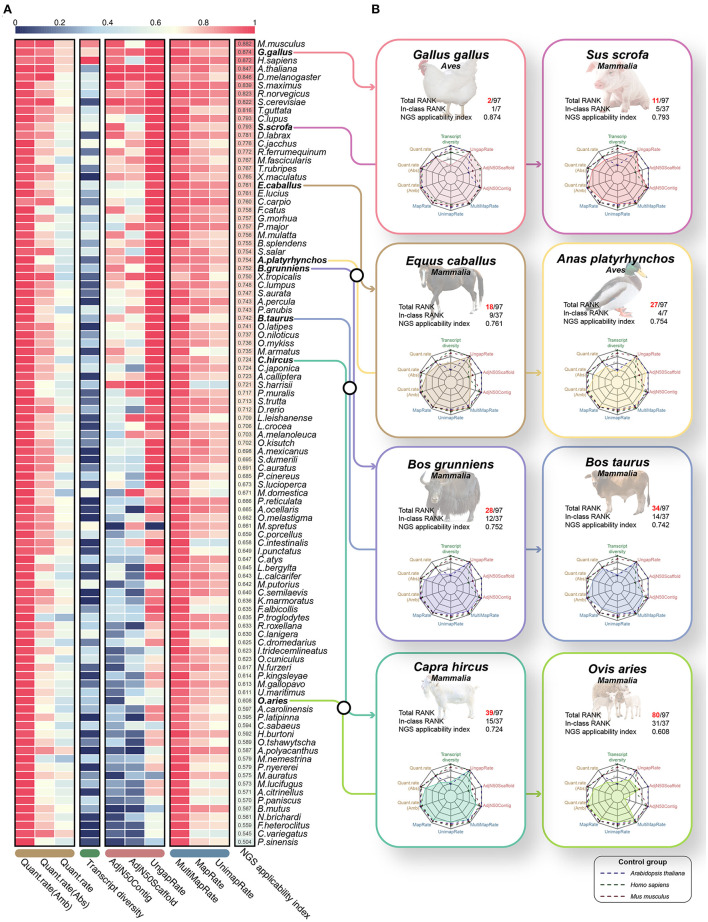
Quality evaluation results of 97 species through the proposed NGS applicability index based on the 10 quality evaluation indicators verified through this study. **(A)** Heatmap for a total of 10 quality evaluation indicators selected through this study. The heatmap includes three assembly evaluation indicators and three performance indicators derived from the mapping process, which can relatively evaluate the quality of genomes. In addition, transcript diversity and three performance indicators derived from the quantification process are included to relatively evaluate the gene models. Finally, all 97 species were sorted in descending order through the NGS applicability index, which is the result of the weighted sum of these 10 quality evaluation indicators. All values have a scale of 0.0 to 1.0, and the closer to 1, the higher the quality. **(B)** Results of benchmarking quality evaluation of reference genome and gene annotation for 8 livestock animals.

Finally, we proposed the NGS applicability index by integrating 10 validated effective indicators that can evaluate the reference genome and gene annotation (Figure 6A, Supplementary Table 11). As a result, mice (0.882), chickens (0.874), humans (0.872) and *Arabidopsis* (0.847) species were observed in the order of highest scores (Figure 6A), which revealed that the NGS applicability index is valid for relative quality assessment in diverse species. We expected that through this NGS applicability index, we could evaluate the boundaries of NGS application research and the direction of development to improve the quality of the genome and gene annotation for a specific species. For example, although *Arabidopsis* and turbot showed extremely high NGS applicability indices, transcript diversity was 0.233 and 0.46, respectively, compared to other high-ranking species. From this, there is no technical problem in performing applied NGS technologies, such as whole genome resequencing or RNA-seq, but it is not possible to study various types of transcripts, including lncRNAs and various small ncRNAs. Simultaneously, it can be understood that these species will improve the direction of increasing the transcript diversity of gene annotations, such as diverse ncRNAs. An integrated quality index of 0.751 on average was observed in all eight livestock animals, it has not yet reached the level of other model animals except for chickens, suggesting that it has stable quality compared to other species (Figure 6B). Because relatively low quantification success rates are observed in goats, yaks, and sheep, gene annotation must be improved soon.

**Figure 7:**
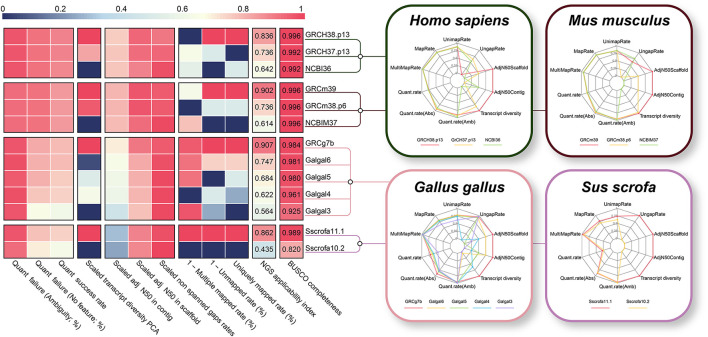
Technical validation of the NGS applicability index based on the different genome builds. Comparison between NGS applicability index and BUSCO completeness for different version of reference genome and gene annotation. Polygonal charts represent values for each of the 10 effective indicators that make up the NGS applicability index. The larger the polygon area represent the higher the NGS applicability index.

Generally, when an assembly build is upgraded, a significant increase in the quality of the reference genome and/or gene annotation is expected. Taking this into account, we additionally compared different assembly builds from four species with a high NGS applicability index (human, mouse, chicken, and pig) to verify the validity of the proposed NGS applicability index. As expected, as the genome build increased in all four species, the NGS applicability index improved significantly (Figure 7), which is direct evidence supporting the validity of our proposed quality indicator. The BUSCO completeness, a representative methodology for evaluating genome assembly, also showed a tendency to increase as the assembly build improved, but it was observed that the difference was relatively insignificant. In particular, the NGS applicability index showed a clear increase in the order of 0.624, 0.745, and 0.912 for the mouse, but the BUSCO Completeness was the same at 0.996. This result is direct evidence that the NGS applicability index can show higher quality assessment discernment by simultaneously considering more diverse aspects than the BUSCO method, which focuses only on the completeness of genome assembly (Supplementary Figure 5).

## Discussion

To date, various studies have been conducted to compare and evaluate the quality of genomes and gene annotations; however, most have been used to compare evolutionarily close species (10, 43) or assembly methods (44, 45). Since most studies have aimed at comparing adjacent minority species, the quality evaluation indicators that have been used are limited, and discussion on the methodology to compare genomes and gene annotations of multiple species is lacking. However, reference genomes and gene annotations are essential data for various NGS application technologies, including RNA-seq data, and have been known to directly affect the performance of essential steps, such as alignments and quantification processes (28, 31). While the application of NGS technology in various species is becoming increasingly common, the quality of these key data can influence the accuracy of the research outcome itself; therefore, it must be evaluated. In this study, genomes and gene annotations of 114 species, including eight livestock species, were obtained from the Ensembl database, and 3,420 RNA-Seq data were collected to attempt diversified quality evaluation in various species (Figure 1). We conducted research to find novel effective indicators for quality assessment, and to select effective indicators among existing quality assessment indexes that can objectively evaluate the genome and gene annotation of a specific species.

Among the indicators generated in the *de novo* assembly process, which is used for quality evaluation of reference genomes, the validity of the N50 values of contig and scaffold levels was first examined (Figure 2). This N50 value, called the contiguity index, refers to the length at which contigs or scaffolds are sorted in length order and reach 50% of the target length of the complete assembly (20). However, this value fluctuates depending on the final target length; therefore, it is not suitable for comparing multiple species with different genome lengths (46). Therefore, in most studies using the N50 index, the genome size of the target species is usually unknown, and has been used to compare the quality of the genome assembly by estimation based on the genome size of evolutionarily close species (21, 22). Because the genome sizes were fixed for the purpose of our study, we converted the N50 value to an effective index that can be compared between multiple species by correcting it with the genome size of the species. As a result, we identified an association with the quality index that directly indicates the quality of the reference genome, such as the number of gaps in the genome (Figure 2A). This gap is the primary target in all reference genome construction studies, and various attempts have been made to minimize it (23–25). We additionally assumed that the repeat elements spread on the genome could be considered as quality indicators; however, the distribution of repeat elements is determined by the characteristics of the species (47) and thus could not be employed as another objective quality indicator (Figures 2E, F). Going one step further, we demonstrated that the three selected genome quality evaluation indicators directly affected the mapping stage of the actual NGS application (Figure 3A). In addition, the genome quality of various species can be evaluated from another perspective through the MQI score, which was created by composing indicators empirically obtained in the mapping step, such as alignment success and failure rate, and failure rate due to multiple mapping (Figure 3C). In conclusion, we selected adjusted N50 values in contig and scaffold levels, number of spanned gaps, and MQI, which are effective indicators for evaluating the quality of reference genomes of various species.

Multiple methods exist for measuring the quality of a reference genome, but the only way to measure the completeness of annotated transcripts in the genome is to compare them with the annotations of evolutionarily similar species (18, 48). In other words, because there is no objective indicator for the quality evaluation of gene annotation, it was not possible to evaluate the quality of various species. In this context, we proposed a novel metric, transcript diversity, to evaluate the completeness of gene annotation in various species (Figure 4). We calculated the diversity of this transcript under the assumption that gene models frequently developed by multiple scientists, such as humans or mice, would eventually be of the highest quality. As evidence for this, we demonstrated that gene annotations in humans and mice are fine-grained for lncRNAs (Figure 4E), various small RNAs, and pseudogenes (Figure 4G). In the past, the elucidation of protein-coding genes has been a major goal, even in representative gene models, including humans and mice (49). However, as it was revealed that non-coding genes such as various types of lncRNA (50), snRNA (51), snoRNA (52), scaRNA (53), and miRNA (54) are also involved in various functions in living organisms, more diverse transcript types have been included in gene annotation of human and mouse. Considering the developmental history of this representative gene model, we believe that our newly proposed transcript diversity has sufficient value as a new index to measure the quality of gene annotation. In addition, we showed that transcript diversity, a qualitative quality indicator, was independent of QQI, a quantitative quality indicator of gene annotation (Figure 5A). Like the MQI, an empirical quantitative index that can evaluate the quality of the genome in the mapping stage, we proposed QQI as a novel indicator, which can evaluate the quality of gene annotation in the quantification process. We demonstrated that the success rate of quantification of mapped reads and both failure rates depended on the complexity of each gene annotation (Figure 5). This is strong evidence to show that the QQI, which is the sum of these three empirical indicators, is also an indicator that can evaluate gene annotation from a different perspective than the transcript diversity index (Figure 5F). In conclusion, we present a novel transcript diversity index, a qualitative index that can evaluate the gene annotations of various species, and the QQI, a quantitative index that can be empirically evaluated. We also demonstrated that they can be used to evaluate the quality of gene annotation in diverse species.

In this study, we attempted a novel approach to compare the quality of reference genomes and gene annotations of multiple species; however, there were limitations. First, we limited the number of species to those from which could collect more than 30 samples of RNA-seq data from species listed in the Ensembl database. If additional species are considered , there is a possibility that the evaluation of the middle and lower ranks may change. Second, although quality control was performed as best as possible for the 30 RNA-seq data samples collected for each species, the data contained random errors, as experimentally identical tissues and environmental conditions were not controlled across all species. This factor can affect the empirical quality metrics. Third, only an intuitive scaling method incorporating 10 quality evaluation indicators was applied in this study. We believe that a methodology that can efficiently integrate heterogeneous indicators derived from these diverse species will be elucidated in near future. Lastly, we considered only those quality evaluation indicators that could be obtained from available data; information that was not publicly available, such as the mis-assembly rate or assembly depth coverage, could not be considered. However, because the relative methodology proposed in this study is a framework, these practical issues are expected to be automatically resolved as reference genomes and gene annotations for various organisms are revealed. Concurrently, the relative index will become more accurate.

## Data availability statement

The original contributions presented in the study are included in the article/Supplementary material, further inquiries can be directed to the corresponding author.

## Author contributions

SPar, JiL, and MS designed the study and wrote the manuscript. SPar, JiL, JK, DK, and MS analyzed the data. SPar and JiL collected sequencing data. All authors reviewed and edited the manuscript, contributed to the article, and approved the submitted version.
