# Supplementary material for: Benchmark study for evaluating the quality of reference genomes and gene annotations in 114 species
Source: Front Vet Sci. 2023 Feb 21;10:1128570. doi: 10.3389/fvets.2023.1128570 (PMC9988948; doi:10.3389/fvets.2023.1128570)
Supplement: Supplementary file 1 [file Data_Sheet_1.PDF]

Supplementary Material

Supplementary Figures

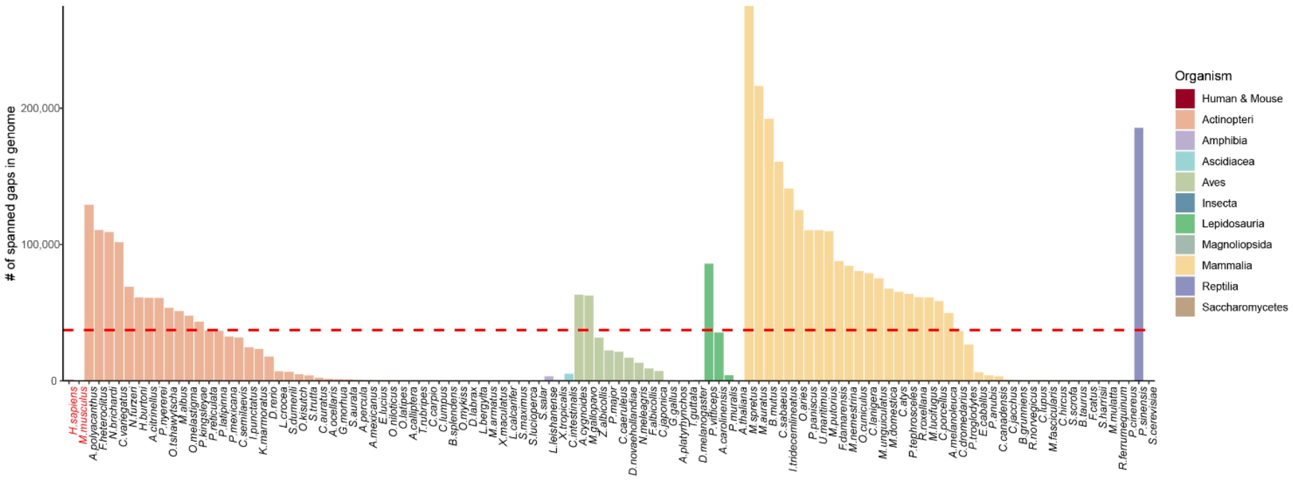

Supplementary Figure 1. The number of spanned gaps in 109 species. Red dotted line represents average spanned gaps across the 109 species.

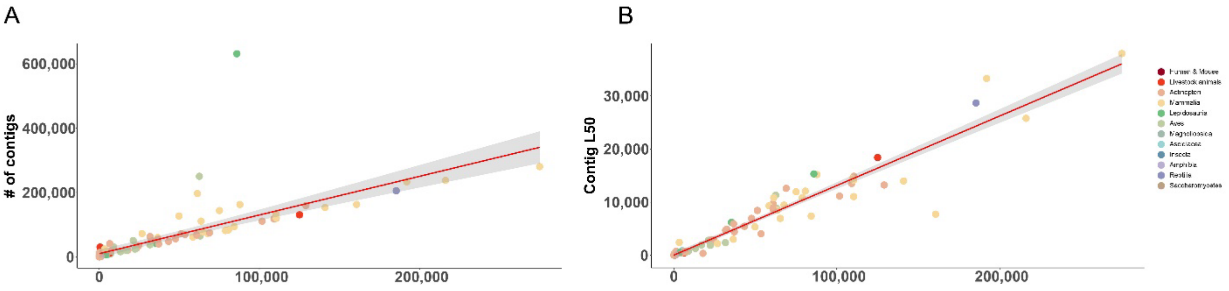

Supplementary Figure 2. Correlation between number of spanned gaps and number of contigs and contig L50.

The red lines are the linear fitted lines, and the standard errors are shaded in grey. **(A)** Comparison of number of spanned gaps and number of contigs in 109 species. A correlation of 0.751 was observed between the two variables. **(B)** Relationship between number of spanned gaps in genome of 109 species and contig L50. A correlation of 0.959 was observed between the two variables.

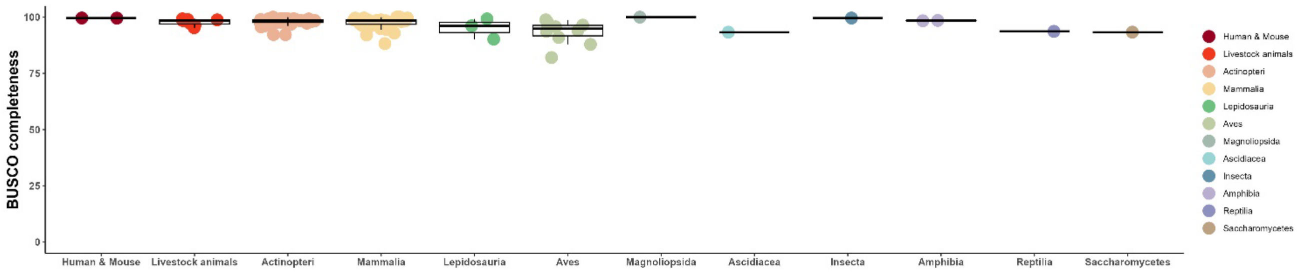

Supplementary Figure 3. The BUSCO completeness in 109 species

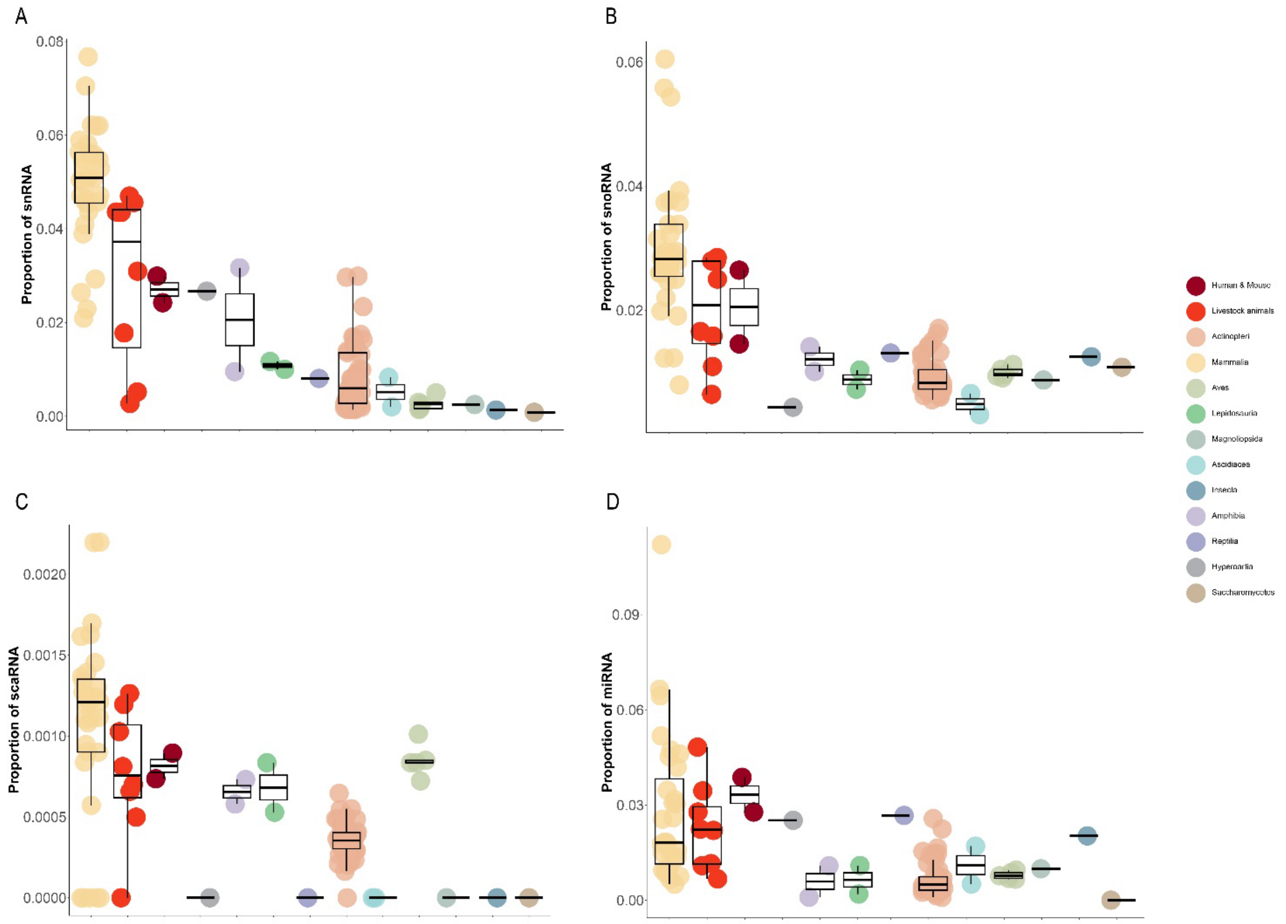

Supplementary Figure 4. Proportion of small noncoding RNAs in 102 species.

(A-D) was proportion of snRNA, snoRNA, scaRNA and miRNA**, respectively**.

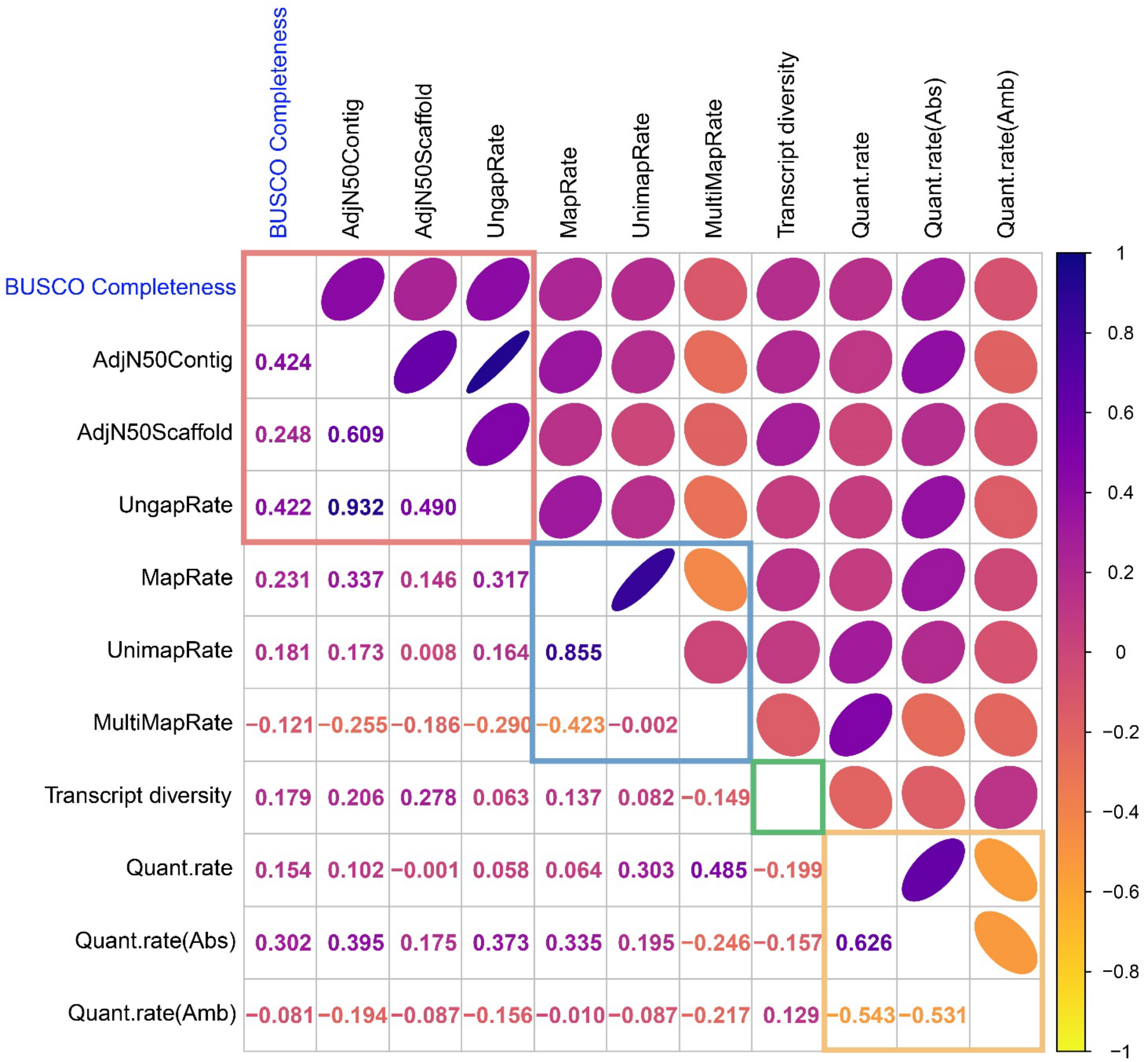

Supplementary Figure 5. Relationship between 10 effective indicators and BUSCO completeness. BUSCO completeness showed high Spearman's correlation with genome assembly statistics of 10 effective indicators.
